# Memantine has no effect on K_ATP_ channels in pancreatic β cells

**DOI:** 10.1186/s13104-018-3715-9

**Published:** 2018-08-25

**Authors:** Ryota Imai, Shingen Misaka, Shoichiro Horita, Shoko Yokota, Rie O’hashi, Yuko Maejima, Kenju Shimomura

**Affiliations:** 1 Department of Bioregulation and Pharmacological Medicine, Fukushima Medical University School of Medicine, Fukushima, Japan; 2 Tsumura Kampo Research Laboratories, Kampo Research & Development Division, Tsumura & Co., Ibaraki, Japan

**Keywords:** K_ATP_ channel, Memantine, Pancreatic β cells, Alzheimer’s disease, Diabetes, Insulin secretion

## Abstract

**Objective:**

Memantine, a drug for Alzheimer’s disease, is considered to suppress excessive stimulation of *N*-methyl-d-aspartic acid receptors and to prevent neuronal death. However, a recent report indicated that the neuronal K_ATP_ channel also can become a target of memantine. The K_ATP_ channel is a key regulator of insulin secretion in pancreatic β cells. Therefore, if memantine could inhibit the K_ATP_ channel in pancreatic β cells, it would be an effective drug for both Alzheimer’s disease and diabetes. However, there is no report on the effect of memantine on the K_ATP_ channel in pancreatic β cells. Therefore, we investigated whether memantine affect the blood glucose level, insulin secretion and K_ATP_ channel activity in pancreatic β cells.

**Results:**

An intraperitoneal glucose tolerance test was performed with or without memantine (1 mg/kg) injection in intact mice. Insulin secretion from isolated islets was measured under low (2 mM) and high (20 mM) glucose concentrations with or without memantine (1 μM). The effect of memantine (1 μM) on K_ATP_ channel currents in isolated pancreatic β cells was recorded using the whole-cell patch-clamp technique. Memantine had no effect on the blood glucose level, insulin secretion from isolated islets or K_ATP_ channel current in pancreatic β cells.

## Introduction

The growing prevalence of diabetes has become a global health concern [1]. Diabetes is known to cause complications such as retinopathy, nephropathy neuropathy, and also known to induce cardiovascular diseases and cerebral infarction. Furthermore, diabetes has been reported to be a risk factor for developing dementia [2].

As with diabetes, the number of dementia patients is also growing [3, 4]. Alzheimer’s disease is considered as the main cause of dementia. Memantine, an *N*-methyl-d-aspartic acid (NMDA) receptor antagonist, was approved in 2011 as one of the drug options for the treatment of Alzheimer’s disease [5]. It is considered to improve the symptoms of Alzheimer’s disease by suppressing excessive stimulation of the NMDA receptor and enable to transmit appropriate signals that lead to the prevention of neuronal death [6]. Memantine is also reported to improve learning impairment and cognitive function [7, 8].

K_ATP_ channel is a potassium channel composed of Kir6.x (Kir6.1 and Kir6.2) and sulfonylurea receptor (SUR; SUR1, SUR2A and SUR2B) subunits, and is sensitive to adenosine triphosphate (ATP) [9–15]. In pancreatic β cells, with the rise of blood glucose, ATP produced by glucose metabolism inhibits the K_ATP_ channel, leading to membrane depolarization and calcium influx through voltage-dependent calcium channel, ultimately inducing insulin secretion [16–22]. The K_ATP_ channel is also expressed in the cerebral cortex, hypothalamus and hippocampus in the brain [23, 24].

A recent report by Moriguchi et al. [25] showed that memantine can inhibit the K_ATP_ channel in the neuron of hippocampus and improve cognitive deficits in mouse model of Alzheimer’s disease. Because this report showed that memantine can block the K_ATP_ channel in the neuron of hippocampus which is composed of the same subunits as that in pancreatic β cells (Kir6.2 and SUR1), it may be possible that memantine can induce insulin secretion. Since diabetes is known to be a risk factor for developing Alzheimer’s disease, memantine may become a drug to treat both Alzheimer’s disease and diabetes. However, there has yet to be a report on effect of memantine on the K_ATP_ channel in pancreatic β cells.

In this study, we investigated effects of memantine on blood glucose level, insulin secretion and K_ATP_ channel activities in pancreatic β cells.

## Main text

### Materials and methods

#### Animal

A total of 35 male C57BL/6J mice purchased from Japan SLC (Shizuoka, Japan) were used in this study. The animals were housed on a 12 h light/dark cycle (07:00–19:00) with conventional food and water ad libitum. At the end of experiments, all animals were disposed with euthanasia under supervision of the Fukushima Medical University Institute of Animal Care and Use Committee. All experiments were approved by the Fukushima Medical University Institute of Animal Care and Use Committee.

#### Glucose tolerance test

Intraperitoneal glucose tolerance test (IPGTT) was performed as described previously [26]. On the day of the experiment, food was deprived at 09:00 and IPGTT (2 g/kg) began at 13:00 with and without memantine (1 mg/kg) dissolved in glucose solution. Blood was sampled by cutting the surface of the tail skin. Blood glucose levels were measured using Glucocard (Arkray, Kyoto, Japan).

#### Measurement of plasma memantine concentration

Thirty minutes after the intraperitoneal administration of memantine (1 mg/kg), mice were sacrificed and blood samples were collected. Plasma samples (25 μL) were diluted with 20 mM ammonium acetate (pH 6.0) and applied to solid-phase extraction column (EVOLUTE CX EXPRESS, 30 μm, 30 mg/1 mL, Biotage, Uppsala, Sweden). After washing with 50 mM ammonium acetate and methanol, samples were eluted with 1 mL of methanol/ammonium hydroxide (95:5, *v/v*). The eluent was dried up with N_2_ gas at 40 °C, followed by reconstitution with 50 μL borate buffer (pH 8.5). For fluorometric measurement of memantine, 50 μL of 20 mM 4-fluoro-7-nitro-2,1,3-benzodiazole in acetonitrile was added and incubated for 5 min at 60 °C in the dark. The reaction was stopped by addition of 50 μL of 0.1 M HCl on the ice. After filtration through a 0.22-μm filter membrane (Millex-LG, Millipore, Bedford, MA, USA) into a 96-well plate, 10 μL of the residual solution was injected to the ultra performance liquid chromatography system (Waters, Milford, MA, USA). Chromatographic separation was performed using AQCUITY BEH C18 column (50 mm × 2.1 mm, particle size 1.7 μm, Waters) at 40 °C under isocratic condition. The mobile phase consisted of water and acetonitrile (35:65, *v/v*) with a flow rate of 0.5 mL/min. NBD-memantine was detected with the excitation wavelength set at 470 nm and the emission at 540 nm.

#### Insulin secretion

Islets were isolated by liberase digestion, and cultured overnight in DMEM medium. After 1 h of starvation in 2 mM glucose, insulin secretion was measured by static incubation (5 islets/well) in 1 mL of test solutions of Krebs–Ringer buffer with and without memantine (1 μM). The Krebs–Ringer buffer contained (in mM) 118.5 NaCl, 2.54 CaCl, 1.19 KH_2_PO_4_, 4.74 KCl, 25 NaHCO_3_, 1.19 MgSO_4_, and 10 HEPES (pH 7.4 with NaOH) with 0.1% bovine serum albumin. Islets were transferred into ethanol/acetic acid solutions (95:5, *v/v*) for insulin content measurements. Insulin was measured using ELISA assay kit (Morinaga, Yokohama, Japan), and secreted insulin was expressed as a percentage of contents.

#### Electrophysiology

Electrophysiological experiments were performed as previously described [26–28]. Islets were dispersed into single cells and maintained in RPMI medium at 37 °C in a humidified atmosphere (5% CO_2_) and used within 1–2 days. All electrophysiological measurements were performed at room temperature (22–25 °C) using an EPC-800 patch-clamp amplifier (HEKA, Lambrecht/Pfalz, Germany) and pCLAMP 10 software (Molecular Devices, CA, USA). The pipette solution contained (in mM) 107 KCl, 2 MgCl_2_, 1 CaCl_2_, 10 EGTA, 10 HEPES, and 0.3 ATP (pH 7.2 with KOH), and the extracellular solution contained (in mM) 138 NaCl, 5.6 KCl, 1 MgCl_2_, 10 HEPES, and 2.6 CaCl_2_ (pH 7.4 with NaOH). The effects of memantine (1 μM) on K_ATP_ channel currents were evaluated using the standard whole-cell technique by applying a holding potential of − 70 mV with ± 10 mV steps at a duration of 250 ms. Data were analyzed using Clampfit software (Molecular Devices).

#### Statistical analysis

The statistical significance of the data was assessed using two-way repeated measures ANOVA followed by Bonferroni’s post hoc test, student’s *t*-test and paired *t*-test. All data were expressed as mean ± SEM.

### Results

#### Plasma concentration and effect of memantine on blood glucose level

**Fig. 1:**
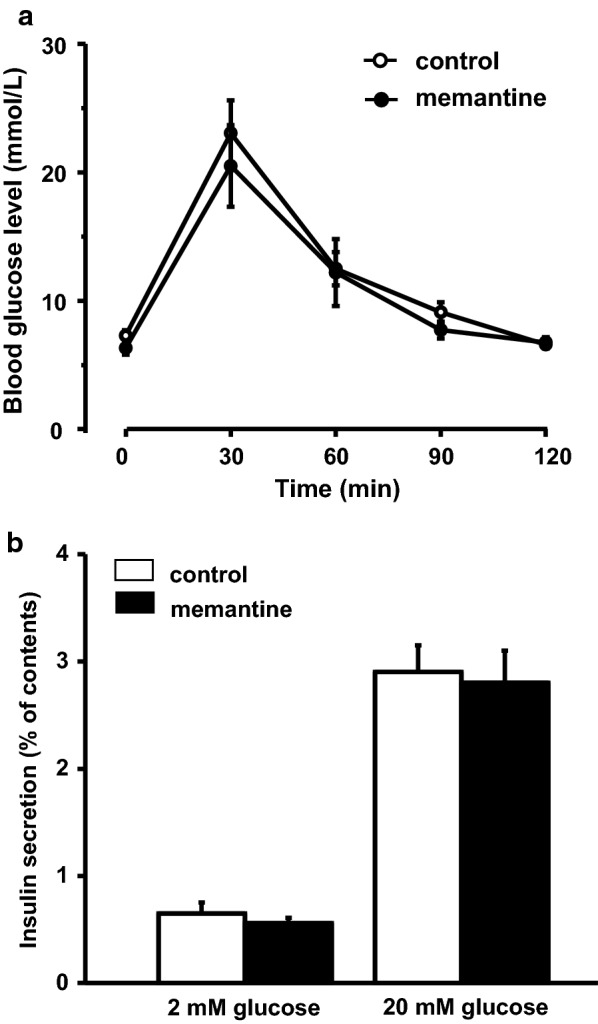
The effects of memantine on blood glucose level and insulin secretion in pancreatic β cells. **a** Change of blood glucose level in IPGTT with or without memantine (1 mg/kg: n = 10 mice each). **b** The amount of insulin secretion from isolated islets in the presence of glucose (2 mM and 20 mM) after application of memantine (1 μM: n = 12 − 18 wells for each condition with islets collected from 6 mice)

In order to investigate the effect of memantine on blood glucose, we conducted IPGTT for 120 min with and without intraperitoneal administration of memantine (1 mg/kg) in mice (n = 10 for each group). Measurement of plasma memantine concentration showed 0.53 ± 0.04 μM (n = 5 mice) at 30 min after intraperitoneal injection of memantine (1 mg/kg). Study by Moriguchi et al. administered memantine orally and did not measure the plasma concentration. However, based on pharmacokinetics study of memantine administration in mice by Beconi et al. [29], oral and peripheral administration of memantine (1 mg/kg) show similar plasma concentration course up to 8 h. Therefore, we considered our intraperitoneal memantine administration would be similar condition to experiments performed by Moriguchi et al. [25] and administered same amount of memantine peripherally. The blood glucose level in the control group increased with a peak at 30 min and gradually returned to the normal level at 120 min. This blood glucose level in memantine treated mice also showed the same tendency with no significant difference compared to that in the control group (two-way repeated measures ANOVA followed by Bonferroni’s post hoc test: Fig. 1a).

#### Effect of memantine on insulin secretion from isolated islets

Next, we measured the amount of insulin secretion after memantine application using isolated islets. Because serum concentration of memantine was 0.53 ± 0.04 μM, slightly higher concentration (1 μM) memantine was applied to islets for this study. The glucose concentration of 2 and 20 mM were used to represent fasting and postprandial blood glucose level. Memantine did not affect the insulin secretion from pancreatic β cells in both glucose concentrations (student’s *t*-test: Fig. 1b).

#### Effect of memantine on K_ATP_ channel current of β cells

**Fig. 2:**
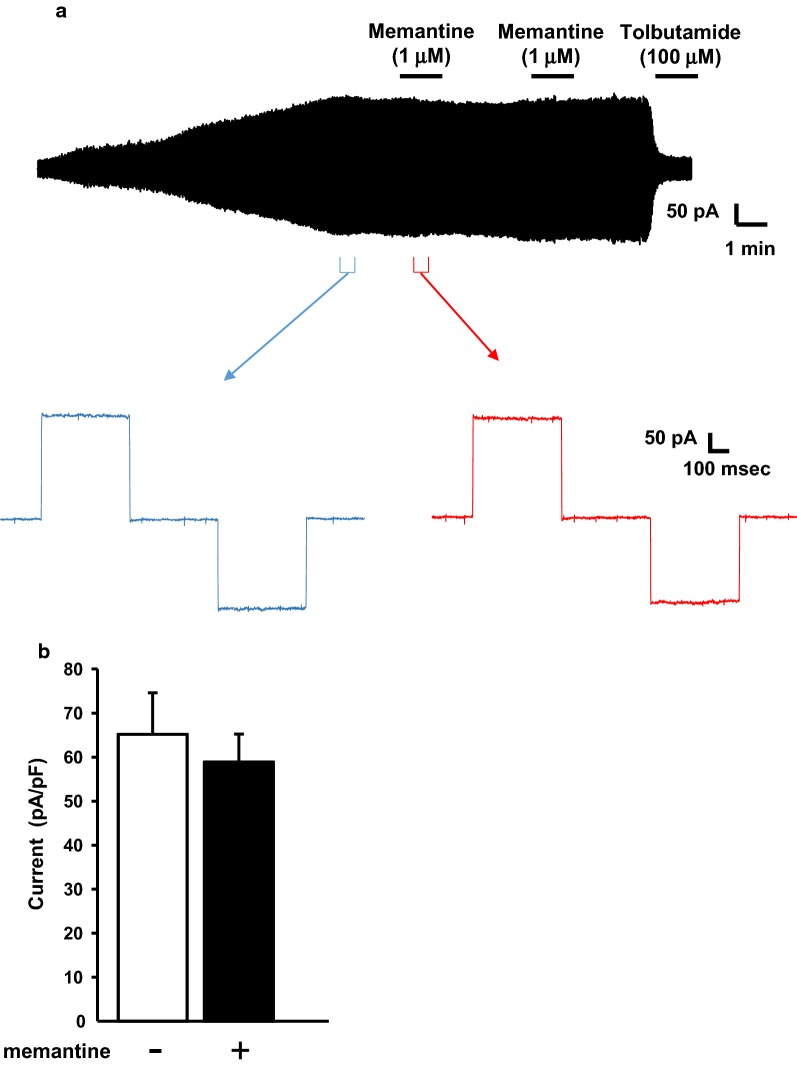
The effects of memantine on K_ATP_ channel current in pancreatic β cells. **a** Top trace shows the full representative K_ATP_ channel currents recording from pancreatic β cells with application of memantine (twice). Bottom traces show enlarged K_ATP_ channel currents before (blue) and after (red) application of memantine (1 μM) during a holding potential of − 70 mV with ± 10 mV steps at a duration of 250 ms. **b** The bar graph showing the summary of K_ATP_ channel currents of pancreatic β cells before and after application of memantine (n = 5 cells from 4 mice)

We recorded the K_ATP_ channel current of pancreatic β cells isolated from intact mice using the whole-cell patch-clamp technique. Application of 20 mM glucose had no effect on K_ATP_ channel current, indicating that the intracellular complex, such as the glycolysis system, is replaced by pipette solution. As shown in top trace of Fig. 2a, memantine (1 μM) was applied twice after current level stabilized in maximum level after washout of ATP. Memantine failed to inhibit the K_ATP_ channel in both initial and second application (paired *t*-test: Fig. 2). The current recorded in this study was confirmed to be K_ATP_ channel current by applying selective blocker of K_ATP_ channel (100 μM tolbutamide: Fig. 2a top).

### Discussion

Based on a report by Moriguchi et al. [25], we hypothesized that memantine may inhibit the K_ATP_ channel in pancreatic β cells, increase insulin secretion and decrease blood glucose level. However, our results indicated that memantine has no effect on the K_ATP_ channel in pancreatic β cells, insulin secretion or blood glucose level.

As with present study, memantine has been reported to have no effect on blood glucose level and serum insulin concentration in normal mice [30]. However, other reports have shown that memantine increased serum insulin and reduced blood glucose level in diabetic and ob/ob mice [25, 30]. It is reported that in diabetic mouse and human, plasma glutamate concentration is higher compared to non-diabetics [30]. The NMDA receptor is expressed in pancreatic β cells and its inhibition is reported to inactivate the K_ATP_ channel, which ultimately improves blood glucose level in a type 2 diabetes mouse model [31–33]. Therefore, it is more likely that memantine improved the diabetic condition by inhibiting excessive activation of the NMDA receptor in pancreatic β cells rather than affecting K_ATP_ channel activity. This is further supported by the fact that dextromethorphan, a noble NMDA receptor inhibitor, showed insulin secretion promoting action and blood glucose lowering effect in db/db mice and type 2 diabetes patients [31, 34]. Because we used non-diabetic mice for the experiments, the blood glutamate concentration and activity of NMDA receptor in pancreatic β cells may have been normal. This may explain why memantine did not influence the blood glucose level or insulin secretion in this study.

In the electrophysiological study, we have shown that memantine had no effect on K_ATP_ channel activity in pancreatic β cells. Because the application of 20 mM glucose did not affect K_ATP_ channel current in our method [35], the K_ATP_ channel current recorded in this study should reflect the direct K_ATP_ channel activity. Therefore, it can be considered that memantine has no direct effect on the K_ATP_ channel in pancreatic β cells. However, aside from a report by Moriguchi et al., Giustizieri et al. [36] also showed that memantine can reduce neuronal K_ATP_ channel conductance in dopamine neurons of substantia nigra pars compacta. Our present data only show that memantine has no effect on K_ATP_ channels of pancreatic β cells and therefore neuron K_ATP_ channels may be regulated in different manner. It is possible to speculate that memantine may inhibit the K_ATP_ channel through another factor that is present in the neuron but not in pancreatic β cells. So far there is no other study that investigated effect of memantine on pancreatic K_ATP_ channel. Further studies are required to elucidate the factor that induces K_ATP_ channel inhibition by memantine in neurons.

### Conclusions

We showed that memantine has no effect on K_ATP_ channel in pancreatic β cells. Memantine may induce K_ATP_ channel inhibition in neurons through factors that are not in pancreatic β cells. However, it is important to note from clinical point of view that this study examined the effect of single dose memantine treatment on insulin secretion. The change of plasma memantine concentration is not experimentally established in different species which may have different pharmacokinetics than human. Further studies are required.

## Limitations

- We were unable to identify the factors that inhibit K_ATP_ channel activity in neurons.
- Effect of NMDA receptor inhibition on insulin secretion is not investigated in this study.

## Abbreviations

NMDA: *N*-methyl-d-aspartic acid
SUR: sulfonylurea receptor
ATP: adenosine triphosphate
IPGTT: intraperitoneal glucose tolerance test
